# Human metabolome variation along the upper intestinal tract

**DOI:** 10.1038/s42255-023-00777-z

**Published:** 2023-05-10

**Authors:** Jacob Folz, Rebecca Neal Culver, Juan Montes Morales, Jessica Grembi, George Triadafilopoulos, David A. Relman, Kerwyn Casey Huang, Dari Shalon, Oliver Fiehn

**Affiliations:** 1 West Coast Metabolomics Center, University of California, Davis, CA USA; 2 Department of Genetics, Stanford University School of Medicine, Stanford, CA USA; 3 Department of Medicine, Stanford University School of Medicine, Stanford, CA USA; 4 Silicon Valley Neurogastroenterology and Motility Center, Mountain View, CA USA; 5 Department of Microbiology and Immunology, Stanford University School of Medicine, Stanford, CA USA; 6 Chan Zuckerberg Biohub, San Francisco, CA USA; 7 Infectious Diseases Section, Veterans Affairs Palo Alto Health Care System, Palo Alto, CA USA; 8 Department of Bioengineering, Stanford University, Stanford, CA USA; 9 Envivo Bio, San Francisco, CA USA

**Keywords:** Diagnostic markers, Ileum, Metabolomics, Microbiome, Metabolism

## Abstract

Most processing of the human diet occurs in the small intestine. Metabolites in the small intestine originate from host secretions, plus the ingested exposome^1^ and microbial transformations. Here we probe the spatiotemporal variation of upper intestinal luminal contents during routine daily digestion in 15 healthy male and female participants. For this, we use a non-invasive, ingestible sampling device to collect and analyse 274 intestinal samples and 60 corresponding stool homogenates by combining five mass spectrometry assays^2,3^ and 16S rRNA sequencing. We identify 1,909 metabolites, including sulfonolipids and fatty acid esters of hydroxy fatty acids (FAHFA) lipids. We observe that stool and intestinal metabolomes differ dramatically. Food metabolites display trends in dietary biomarkers, unexpected increases in dicarboxylic acids along the intestinal tract and a positive association between luminal keto acids and fruit intake. Diet-derived and microbially linked metabolites account for the largest inter-individual differences. Notably, two individuals who had taken antibiotics within 6 months before sampling show large variation in levels of bioactive FAHFAs and sulfonolipids and other microbially related metabolites. From inter-individual variation, we identify *Blautia* species as a candidate to be involved in FAHFA metabolism. In conclusion, non-invasive, in vivo sampling of the human small intestine and ascending colon under physiological conditions reveals links between diet, host and microbial metabolism.

## Abstract (web summary)

The authors use a non-invasive sampling device to collect intestinal samples in 15 healthy male and female participants and analyse them through mass spectrometry and 16S rRNA sequencing. They identify thousands of biomolecules throughout the intestinal tract, many of which remain unknown and highlight differences between intestinal and stool metabolomes.

## Main

**Fig. 1:**
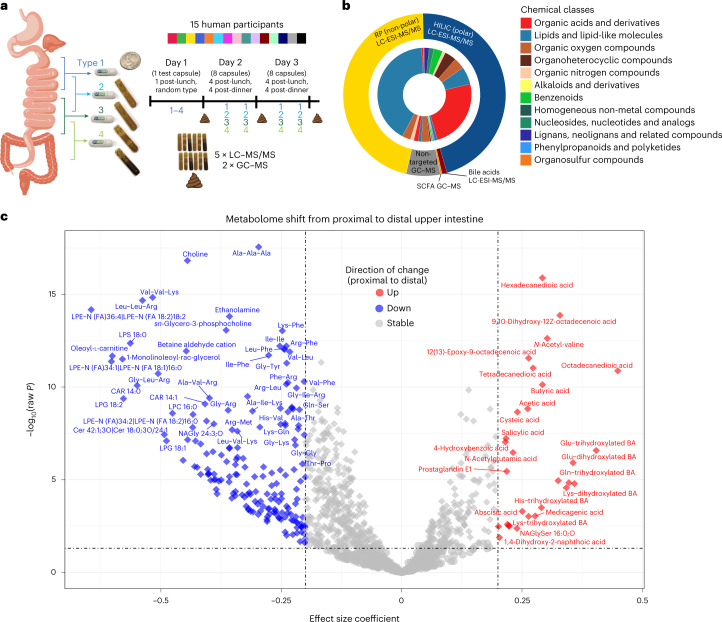
Comparison of proximal and distal upper intestinal metabolite levels reveals significant differences in a wide range of compounds. **a**, Study design for upper intestinal tract investigation. Four types of an intestinal sampling device were used to sample the proximal to distal upper intestines. Fifteen human participants swallowed at least 16 devices over 2 d after lunch and after dinner after an initial test on day 1. Devices were retrieved and analysed by targeted and non-targeted LC–MS/MS and GC–MS methods. **b**, Identified metabolites from the five metabolome assays used to analyse samples. Chemical class fractions are included based on automated ClassyFire chemical classification. **c**, Significance of differences between upper intestinal tract regions was calculated using LMM. Horizontal dashed-dotted line represents the significance threshold *P* < 0.05 (*n* = 1,182 metabolites). Circles indicate non-significance and diamond shapes indicate significance (*P* < 0.05) after FDR correction. Only metabolites detected in >50% of intestinal samples were included in this analysis (*n* = 1,182). Effect size coefficient is the slope estimated by LMM, with positive (negative) coefficient indicating higher (lower) levels in the distal compared to proximal upper intestine. Vertical dashed-dotted lines are ±0.2 effect size coefficient.

We aimed to comprehensively study metabolomic differences among luminal samples from the upper intestinal tract of 15 healthy individuals to better understand the extent of spatial and temporal variation and to gauge the prospects of integrating metabolome and microbiome data. In a related companion publication^4^, we use these devices to study variation along the gut in microbiota composition, prophage induction, the host proteome and microbial modification of bile acids. Volunteers swallowed sets of four sampling devices per sampling time point. These ingestible sampling devices consisted of a collapsed collection bladder capped by a one-way valve in a capsule with a pH-sensitive coating. The four types of devices differed only in their enteric coating, which dissolved at pH 5.5 (type 1), pH 6 (type 2) and pH 7.5 (types 3 and 4) (Fig. 1a). The thickness and pH responsiveness of the coating enabled sampling at specific locations of the intestinal tract after gastric emptying. The devices did not contain any electronics beyond a passive radio frequency identification chip for tracking purposes. Once the coatings dissolved, an elastic collection bladder expanded and collected up to 400 µl of luminal contents through vacuum suction. The one-way valve prevented loss of sample and contamination from downstream fluids. Stool samples were frozen at −20 °C and all devices were recovered from the stool before analysis. Liquid contents were retrieved from devices using hypodermic needles. Aliquots of the raw sample were used for 16S ribosomal RNA microbiome analyses and the supernatants from centrifuged samples were used for metabolomic studies. Here, we perform a meticulous analysis of the metabolome in the same samples, reporting metabolites never before detected in human samples, key biomarkers of diet and comparison of chemical profiles across and within participants (Supplementary Tables 1 and 2).

**Extended Data Fig. 1:**
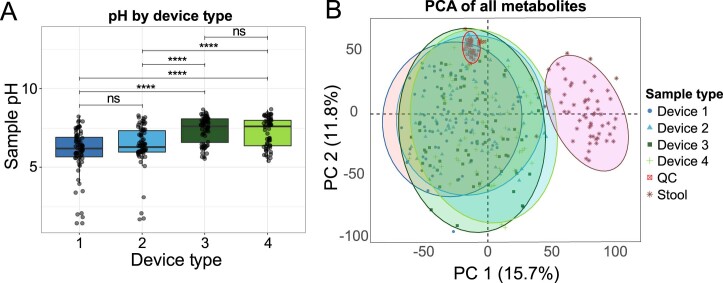
Summary visualizations. **a**, Sample pH for intestinal sampling device types. Boxes represent ±1 interquartile range, and the line extends to the sample furthest from the median sample with a maximum extension of 1.5 interquartile ranges away from the median. All samples are plotted as individual points. (n = 274 total devices, Device type 1 = 75, Device type 2 = 70, Device type 3 = 69, Device type 4 = 60) ****: *p* < 0.0001 (Wilcoxon signed-rank). **b**, Principal Component Analysis (PCA) of normalized peak heights from the metabolomics dataset. QC samples are pooled intestinal contents from human intestinal tract samples.

The measured pH of the luminal contents for device types 1 through 4 was consistent with the expected pH gradient across the intestinal tract^4,5^, covering the duodenum, jejunum, ileum and ascending colon (Fig. 1a). The pH in type 1 and 2 devices was significantly different from type 3 and 4 devices (Extended Data Fig. 1a and Supplementary Table 3; Wilcoxon two-way rank-sum test, *P* = 2.4 × 10^−14^), whereas pH was not significantly different between type 1 and type 2 devices or between type 3 and type 4 devices (Extended Data Fig. 1a). We therefore associated type 1/2 and 3/4 devices with proximal (duodenum and jejunum) and distal (ileum and ascending colon) regions of the upper intestinal tract, respectively.

We used five mass spectrometry assays to analyse the luminal contents captured by these capsule devices and the associated stool samples. By matching chromatographic retention times, accurate precursor masses and mass spectrometric fragmentation (MS/MS) to MassBank.us public and NIST20 licensed libraries, we annotated 1,909 chemicals from gut luminal and stool contents at Metabolomics Standards Initiative confidence levels 1–3 (Supplementary Table 1)^6^, including 155 internal standards used for quality control (QC) and quantification purposes. Additionally, >12,000 unknown chromatographic features were reliably detected above the level of method blanks (Supplementary Table 2). Using ClassyFire software^7^, structurally annotated metabolites fell into 61 chemical subclasses (Supplementary Table 1). Two untargeted high-resolution liquid chromatography (LC) MS/MS assays focusing on hydrophilic and lipophilic metabolites yielded most of the annotated compounds, with 1,612 identifications. Untargeted gas chromatography (GC)–MS added 119 primary metabolites, supplemented by targeting six short-chain fatty acids (SCFAs) and a targeted LC–MS/MS assay for 17 bile acids (Fig. 1b). QC analysis of total metabolic variance revealed separation of stool and intestinal samples, with strong clustering of pooled quality control samples (Extended Data Fig. 1b).

**Extended Data Fig. 2:**
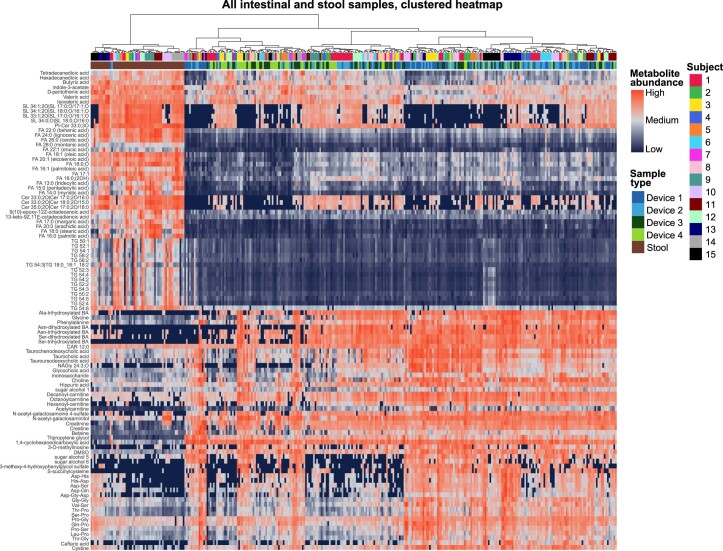
The 80 metabolites most important for distinguishing intestinal device samples from stool samples as determined by partial least square discriminant analysis (PLS-DA). Samples and metabolites are organized on the *x*-axis and *y*-axis, respectively, by hierarchical clustering. Each sample is labeled by subject number and sample type (device type or stool). Color represents metabolite abundance, and the minimum and maximum values are set individually for each metabolite (row).

**Extended Data Fig. 3:**
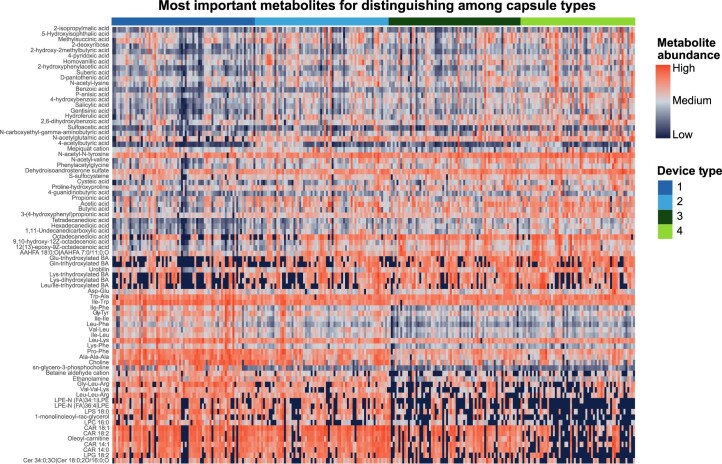
The top 80 metabolites most important for distinguishing device samples by type as determined by partial least square discriminant analysis (PLS-DA). Samples are organized on the *x*-axis by device type, and metabolites are organized on the *y*-axis according to hierarchical clustering. Color represents metabolite abundance, and the minimum and maximum values for color scale are set individually for each metabolite (row).

**Extended Data Fig. 4:**
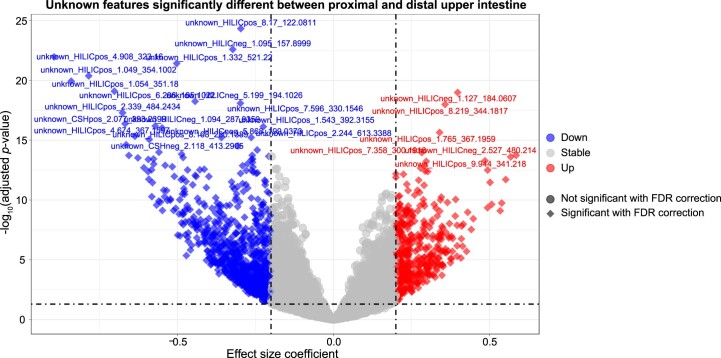
Comparison of levels of unannotated features in the proximal and distal upper intestines. Data from devices 1 + 2 (proximal) compared to data from devices 3+4 (distal). Significance was calculated using a linear mixed effect model (LMM). The horizontal dashed line represents the significance threshold of *p* < 0.05 (For exact *p* values see Supplementary Table 4). Circles indicate metabolites with non-significant differences after false discovery rate (FDR) correction or an effect size coefficient < ± 0.2. Diamonds indicate metabolites with significant (*p*<0.05) differences after FDR correction (*n* = 9,317) and effect size coefficient > ± 0.2. Only features detected in >50% of intestinal samples were included in this analysis (*n* = 9,317 features). Effect size coefficient is the slope estimated by the LMM, with positive (negative) coefficient representing a metabolite that is higher (lower) in the distal compared to proximal upper intestine. Vertical dashed lines are ±0.20 times the effect size coefficient.

Metabolome results revealed notable differences between stool and intestinal samples (Extended Data Fig. 2) and among the intestinal tract samples (Extended Data Fig. 3). To uncover spatial differences across the intestine, we applied linear mixed-effect models (LMMs) that accounted for sampling location (proximal or distal) as well as other variables (Supplementary Tables 3 and 4). Specifically, we studied the 1,182 most prevalent metabolites that were detected in >50% of device samples. Of these, 630 (54%) were significantly different in the proximal compared to distal upper intestine (false discovery rate (FDR) *P* < 0.05; LMM) (Fig. 1c and Supplementary Table 4), with 473 metabolites at higher levels in the proximal compared to distal upper intestine and 157 compounds at lower levels in the proximal compared to distal upper intestine (Fig. 1c). Known microbially generated chemicals including SCFAs^8,9^, secondary bile acids^10^ and some microbially conjugated bile acids^11,12^, increased from the proximal to distal upper intestine (Extended Data Table 1 and Fig. 1c). Of the 11 detected acetylated amino acids, 7 increased from the proximal to distal upper intestine (raw *P* < 0.05; LMM) (Extended Data Table 1 and Fig. 1c). We also examined the 12,346 chemically unannotated metabolite signals, restricting our attention to 9,317 signals that were detected in >50% of intestinal samples (Supplementary File 1). Overall, 3,594 (38%) features were significantly different between the proximal and distal upper intestine, with 1,937 features at higher levels in the proximal compared to distal upper intestine and 1,657 features at lower levels in the proximal compared to distal upper intestine (FDR *P* < 0.05; LMM) (Extended Data Fig. 4).

**Extended Data Fig. 5:**
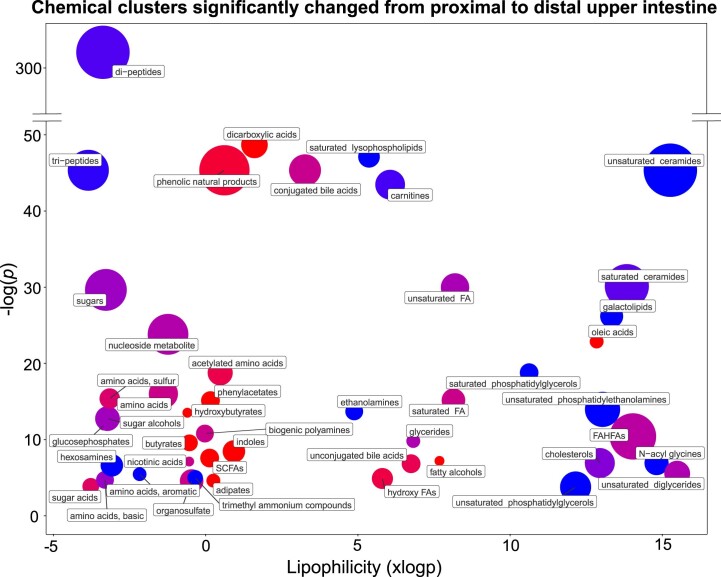
Chemical classes that differed significantly between the proximal and distal upper intestine. Chemical enrichment statistics were performed by ChemRICH. Metabolites detected in >50% of intestinal samples were included in this analysis (*n* = 1182 metabolites). These results were visualized by separating classes by chemical lipophilicity (logP) and chemical class significance level of -log_10_(*p*-value). Red circles indicate that the chemical class was higher in the distal compared to proximal upper intestine, and blue indicates that the chemical class was lower in the distal compared to the proximal upper intestine. Purple indicates the chemical cluster has metabolites that are significantly higher as well as metabolites that are significantly lower in the distal compared to proximal upper intestine. Circle size represents the size of the chemical class.

To interrogate general metabolic differences between locations, we used chemical enrichment statistics. Di- and tripeptides were among the most significantly decreased classes from the proximal to distal upper intestine (Extended Data Table 1 and Extended Data Fig. 5). Of the 333 di- and tripeptides measured, 262 significantly decreased in abundance from the proximal to distal upper intestine (raw *P* < 0.05; LMM) (Extended Data Table 1). Sugars, sugar alcohols, nucleosides, carnitines and ceramides also exhibited significantly higher levels in proximal intestinal tract samples compared to distal samples (Extended Data Table 1 and Extended Data Fig. 5). These spatial differences in the intestine reflect classic digestion and absorption^13^ of di- and tripeptides^14^ and acylcarnitines^15,16^, as well as ceramides that are hydrolyzed to sphingosine and free fatty acids before intestinal uptake^17^. In contrast, SCFAs exhibited increased levels in distal regions (Extended Data Table 1 and Fig. 1c), likely due to their production by microbes^8,9^. Acetylated amino acids, which have been associated with Crohn’s disease^18^, were also at higher levels in the distal compared to proximal upper intestine (Extended Data Table 1 and Fig. 1c), possibly due to slower absorption of acetylated compared to non-acetylated amino acids^19,20^. Bile acids are transformed extensively by microbes and levels of secondary bile acids increased along the intestine^4^. These observations support the notion that the capsule devices sampled from the intended locations. Although average pH levels across devices of a given type also followed the expected trends across the upper intestinal tract, the observed within-person variation in pH for each device type did not correlate with metabolomic changes across distal versus proximal regions.

We measured 28 phenolic metabolites that increased from the proximal to distal upper intestine (Supplementary Table 4). These trends are likely caused by a combination of factors, including enzymatic transformation^21,22^ such as deglycosylation and delayed breakdown of plant cells and cell-wall components by microbial enzymes^23–25^. For example, the flaxseed-affiliated lignan secoisolariciresinol was most significantly enriched in distal compared to proximal samples, likely due to both bioavailability^26^ and deglycosylation^27^.

Dicarboxylic acids also increased in concentration from the proximal to distal upper intestine (Extended Data Table 1 and Fig. 1c). In fact, three of the top six most significantly increased metabolites between the proximal and distal upper intestine were dicarboxylic acids (hexadecanedioic acid, tetradecanedioic acid and octadecanedioic acid) (Fig. 1c). Dicarboxylic acids are generated during catabolism (omega oxidation) of fatty acids, which occurs in human cells^28^, plants^29^ and microbes^30,31^. The lead compound, hexadecanedioic acid, was most strongly correlated with other dicarboxylic acids, plant metabolites, bile acids and known microbially produced compounds (Supplementary Table 5). Epithelial cells contain omega-hydroxylated lipids essential to maintain epithelium barrier function^32^ that can be cleaved by lipases to form dicarboxylic acids. We hypothesize that the consistent and significant increase of dicarboxylic acids along the upper intestine is due to catabolism of human epithelial lipids.

The chemical profiles of intestinal samples differed substantially from those of stool (Extended Data Fig. 2). Thirty-one metabolites were >100 times more abundant on average in the intestine compared to stool. These metabolites consisted of glycinated lipids, sugars, plant natural products, carnitines, microbially conjugated bile acids and *S*-succinylcysteine (Supplementary Table 6). Peptides were also generally at much lower levels in stool samples compared to intestinal samples, especially when compared to the proximal intestine (Extended Data Fig. 2). We also identified >100 metabolites that were >100 times more abundant in stool compared to intestinal samples (Supplementary Table 6); these metabolites were mostly polar lipids such as phosphatidylethanolamines, phosphatidylinositols and phosphatidylglycerols, as well as specific FAHFAs. The high abundance of membrane lipids in stool samples is likely due to the high amount of bacterial cell material in stool compared to luminal samples from the upper intestine.

**Fig. 2:**
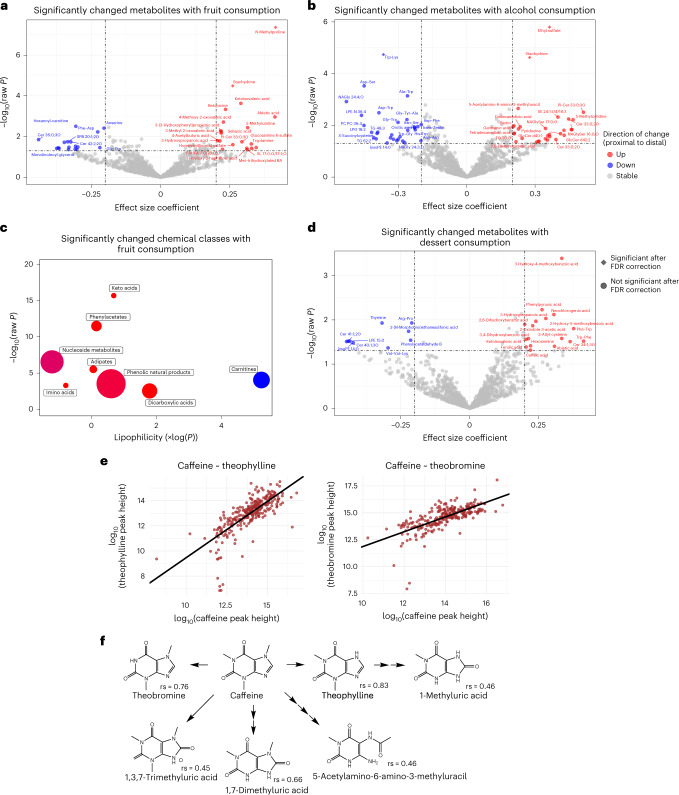
Intestinal metabolite association with food types. **a**,**b**,**d**, Volcano plots show significance of each metabolite to food types of fruit (**a**), alcohol (**b**) and dessert (**d**) calculated by LMM. Consumption is defined as food eaten within 6 h of swallowing sample devices. Significance of *P* < 0.05 (*n* = 1,182 metabolites) is delimited by the lower dashed-dotted horizontal line. Circles indicate non-significance after FDR correction and diamonds indicate significance (*P* < 0.05) after FDR correction (*n* = 1,182). Metabolites detected in >50% of intestinal samples were included in this analysis. Effect size coefficient is the slope estimate calculated by LMM, with positive (negative) coefficient meaning the metabolite was higher (lower) after food consumption. Vertical dashed-dotted lines are ±0.2 effect size coefficient. **c**, Chemical enrichment statistics (ChemRICH) analysis revealed significant chemical classes after fruit consumption visualized by separating classes by chemical lipophilicity (log*P*) and chemical class significance level of −log_10_(*P*). Red circles indicate that the chemical class increased after fruit consumption and blue circle indicates that the chemical class decreased after fruit consumption. Circle size indicates the size of the chemical class. **e**, Theophylline and theobromine levels are strongly associated with caffeine levels. Circles represent measured levels in each sample for which both metabolites were detected. **f**, Chemical diagram of caffeine and known metabolic pathways with structures of detected metabolites and Spearman rank correlation coefficient (rs) for each structure (*P* < 1.0 × 10^−13^ for all metabolites; *n* = 1,182 metabolites).

Next, we used LMM to test for associations of food intake logs recorded by the participants to levels of intestinal tract metabolites. We tested for consumption of fruit, alcohol, dessert, animal protein, vegetables, grains, coffee/tea and dairy food types ingested 6 h before swallowing capsule devices (Supplementary Table 3). After correcting for multiple-hypothesis testing, some food types had no significantly associated metabolites, unsurprisingly due to the small sample size for some food types and strong FDR correction accounting for tests of 1,182 metabolites (Table 1, Fig. 2a–d and Supplementary Table 4). Despite the small size of this study with 15 participants, we were able to validate a range of dietary biomarkers that were previously found in blood and correlated with fruit^33^ and alcohol^34^ consumption, as well as discover other biomarkers not previously identified.

**Table 1:** Participant characteristics

Attribute	Value
Total number of participants	15
Participants completing the study	15
Age	Mean 42, range 22–64
Females	8
Males	7
Antibiotic use within past 6 months	2
Underlying medical conditions	0
Body mass index	Mean 23, range 19–31

Detailed exclusion and inclusion criteria are supplied in Supplementary Table 3.

Using effect size differences of ±0.2 and raw *P* < 0.05, fruit consumption was significantly associated with 20 compounds at increased concentration and 17 metabolites at decreased concentration (Fig. 2a). Some metabolites were directly linked to fruit consumption even with strict FDR-corrected *P* < 0.05 (Fig. 2a), including *N*-methylproline and stachydrine, both of which were previously reported as fruit consumption biomarkers for blood plasma in non-controlled dietary studies^33^. Betonicine, a known component of fruit juice^35^, also increased after fruit consumption at raw *P* < 0.05 (Fig. 2a) but did not achieve the FDR significance threshold. Similarly, three keto acids (4-methyl-2-oxovaleric acid, ketoisovaleric acid and 3-methyl-2-oxovaleric acid) also significantly increased in response to fruit intake at raw *P* < 0.05 (Fig. 2a). Metabolites are not independent of one another, but rather are linked via food compositions and microbial and enzymatic pathways. Therefore, we used ChemRICH chemical set enrichment statistics to identify significantly altered clusters of metabolites (Fig. 2c). This strategy revealed keto acids as the chemical class with the most significant response to fruit (Fig. 2a,c), highlighting keto acids as a fruit biomarker in the human gut. Keto acids are formed from enzymatic deamination of amino acids, carried out in part by gut bacteria^36^. Notably, ChemRICH also revealed that typical fruit ingredients like phenylacetates and phenolic natural products were positively associated with fruit intake (Fig. 2c).

Alcohol consumption was most significantly associated with ethyl sulfate (FDR *P* < 0.05), a known plasma biomarker of alcohol consumption (Fig. 2b)^34^. Stachydrine was linked with both fruit and alcohol consumption (FDR *P* < 0.05). Trp-Lys significantly decreased with alcohol consumption after FDR correction (Fig. 2b). In total, 40 di- and tripeptides decreased with alcohol consumption (raw *P* < 0.05) with a ChemRICH cluster *P* = 8.8 × 10^−18^ (Supplementary Table 4 and Supplementary Figs. 1 and 2). The decrease in di- and tripeptides after alcohol consumption suggested a decrease in total protease activity, possibly due to impaired pancreatic secretion^37,38^ rather than direct inhibition because trypsin and chymotrypsin are active even in 20% ethanol solution^39^. ‘Dessert’ was defined as consumption of high-fat/high-sugar foods, such as soda, cake and ice cream. Two substituted benzoic acids, 3-hydroxy-4-methoxybenzoic acid and 3,4-dihydroxybenzoic acid, were associated with dessert (raw *P* < 0.05) (Fig. 2d). These compounds are metabolic intermediates in the breakdown of vanillin and isovanillin^40^. Neochlorogenic acid was also significantly associated with dessert (raw *P* < 0.05). Neochlorogenic acid is present in a variety of fruits and berries^41^, including cherries^42^ and peaches^43^. Other food types that were included in the mixed-effect model also had significantly associated metabolites (Supplementary Table 4 and Supplementary Figs. 1 and 2).

Caffeine was detected in the majority of samples (Supplementary Table 1). Of note, caffeine was not significantly associated with coffee or tea consumption during the experimental timeframe (FDR *P* = 0.87) (Supplementary Table 4), most likely because caffeine is absorbed rapidly within 1 h of oral intake and has a mean half-life of 4.5 h (range 2.7–9.9 h) in the bloodstream^44^; however, caffeine metabolic pathways were readily discerned through Spearman rank correlation analysis. The six metabolites that were most strongly correlated at FDR *P* < 10^−13^ to caffeine were known caffeine catabolites^45–47^, including theophylline and theobromine (Fig. 2e,f). Caffeine is metabolized and excreted through several routes, including urine^48^ and bile^49^. Bile is the expected origin of caffeine measured in this study, as multiple hours passed between beverage consumption and device-sampling events. While theobromine is known to be present in chocolate, it did not associate with dessert consumption, only with caffeine metabolism. Hence, upper intestinal tract metabolite correlations may enable reconstruction of microbial and enzymatic pathways of exposome metabolism. Dedicated studies across a diverse population using dietary interventions would be needed to associate specific food biomarkers. In addition, as the devices largely preserve bacterial viability^4^, cultured isolates obtained from the devices could be used to confirm individual food metabolome-bacterial interactions.

**Extended Data Fig. 6:**
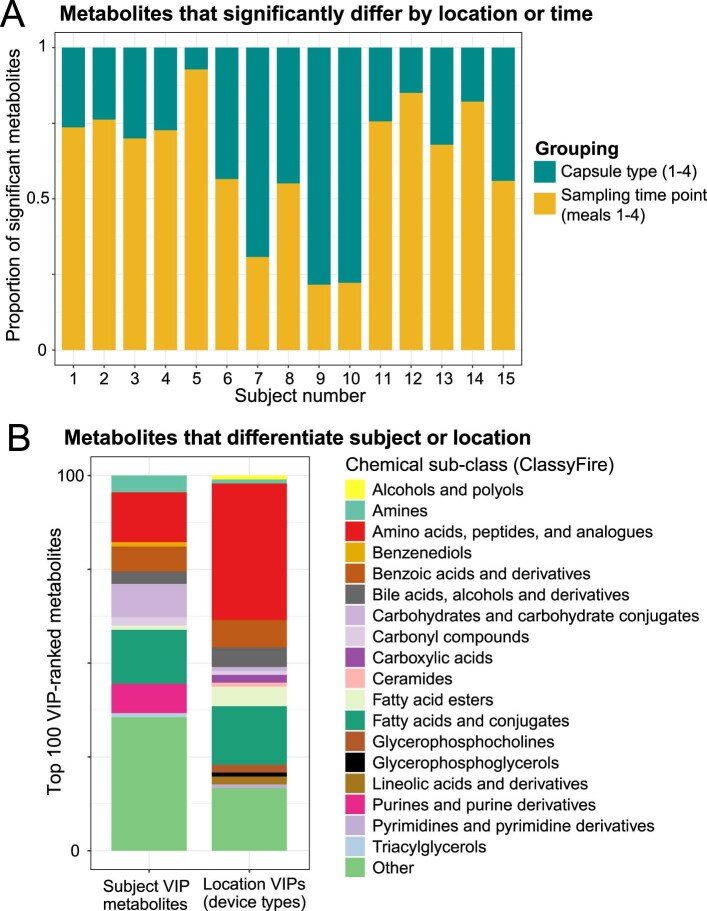
Comparison of temporal and personal variation to spatial variation in the intestinal metabolome. **a**, The number of metabolites that differed significantly different between intestinal regions (device types) or between meals (sampling time points of four capsule devices) calculated for each subject by analysis of variance (Kruskal-Wallis). Only metabolites detected in >50% of samples for each subject were used for this analysis (n = 1182 metabolites). Non-FDR-corrected *p* < 0.05 was used as a significance threshold. **b**, Multivariate discriminant analysis (PLS-DA) was performed to identify metabolites that were most important for distinguishing between subjects, or between regions. The 100 metabolites most important for distinguishing these groups were ranked by variable importance in projection score (VIP) and are categorized by chemical subclass. Chemical subclasses with <3 metabolites are reported as ‘Other’.

**Extended Data Fig. 7:**
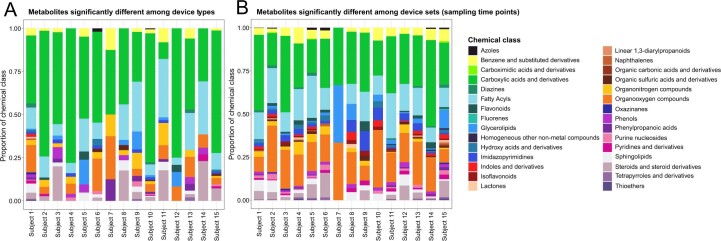
The number of metabolites that were significantly different between intestinal regions (device types) or between meals (sampling time points of sets of 4 devices) were calculated for each subject by analysis of variance (Kruskal-Wallis). Only metabolites detected in >50% of samples for each subject were used for this analysis (n = 1182 metabolites). Non-FDR-corrected *p* < 0.05 was used as a significance value cutoff. **a**, Metabolites with significantly different abundance between intestinal regions for each subject, grouped by chemical class and the proportion of each chemical class. **b**, Metabolites with significantly different abundance between sampling time points, grouped by chemical class and the proportion of each chemical class.

Dietary metabolites were associated with temporal differences during the 2 d and four sampling time points of this study. To investigate whether sampling time or sampling region had a larger impact on upper intestinal metabolites, we used analysis of variance (ANOVA) to calculate the number of metabolites that significantly differed between the four device types by participant, or between the four sampling time points (after each meal) by participant. The large differences in metabolite levels between the proximal and distal sampling regions were often superseded by metabolic differences between time points, showing that 12 of 15 participants had more statistically different metabolites between meals (time points) than between intestinal regions (device types) (Extended Data Fig. 6a,b). A closer inspection of the compound classes that contributed to these differences found that di- and tripeptides (within the chemical class of carboxylic acids) were the largest chemical class that distinguished between device types, representing >70% of all significantly different metabolites in five participants and >40% for another seven participants (Extended Data Fig. 7a). For metabolites that differentiated sampling time points, sugars (organooxygen compounds) were enriched in 13 of 15 participants (Extended Data Fig. 7b). Similarly, more significantly different imidazopyrimidines, indoles and isoflavonoids were found to distinguish sampling time points than intestinal regions (Extended Data Fig. 7). These classes signify dietary metabolites that were different due to variation between food types ingested during different meals, but were not as useful for differentiating between intestinal regions.

Our dataset exhibited large inter-individual variation (Extended Data Fig. 7). As participants were not prescribed specific meals, diet-based variation was expected to differentiate participants and time points. Using multivariate discriminant analysis (PLS-DA), we identified differences in the proportion of metabolites that were most important for differentiating between the proximal and distal intestine (device types) and among the 15 participants (Extended Data Fig. 6b). The large overall variance among samples obscured clear visualization of PLS-DA based on participants, devices or time points. Nonetheless, the 100 metabolites that contributed most to multivariate discrimination revealed participant-specific trends that were best explained by metabolites of plant and microbial origin (Extended Data Fig. 6b), including the pepper compound capsaicin, the flaxseed compound secoisolariciresinol and the microbially produced butyric acid and propionic acid (Supplementary Table 7). Other metabolites that showed participant-specific variation, such as *N*-methylhistamine, phenethylamine, phenylacetaldehyde and succinic acid, may depend on a combination of human, dietary or microbial factors. Notably, hierarchical clustering separated stool samples by participant, whereas intestinal samples did not strongly cluster by participant (Supplementary Fig. 3), perhaps due to the higher abundance of individual-specific gut microbes in the stool relative to the proximal intestine, which was more dominated by the variability in dietary components.

**Fig. 3:**
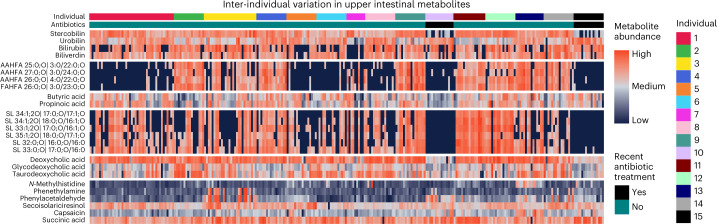
Heat map of metabolites with strong inter-participant differences. Metabolites include bile pigments, fatty acid esters of hydroxy fatty acids (FAHFAs and AAHFAs), SCFAs, sulfonolipids (SLs) and secondary bile acids. Samples are organized by participant and antibiotic consumption is indicated for the two participants who consumed antibiotics 1 and 5 months before this study. Colour of heat map ranges from low (blue) to high (red) of metabolite abundance (peak height) or concentration (in nanograms per millilitre) for bile acids. Minimum and maximum values are used to set colour scale for each metabolite (each row).

**Extended Data Fig. 8:**
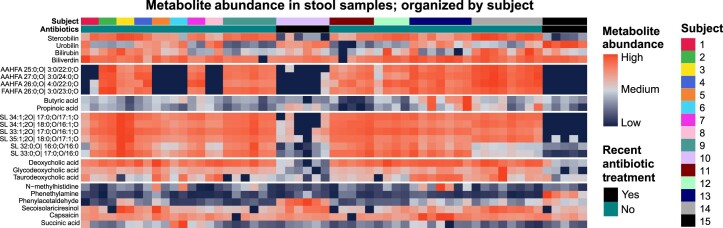
Metabolites with strong inter-subject differences. Metabolites include bile pigments, fatty acid esters of hydroxy fatty acids (FAHFAs and AAHFAs), short chain fatty acids, sulfonolipids (SLs), and secondary bile acids. Data from all stool samples are shown, and samples are organized by subject. The two subjects that consumed antibiotics 1 and 5 months prior to this study are highlighted. Color bar represents metabolite abundance (peak height) or concentration (ng/mL) for bile acids. Minimum and maximum values were used to set the color scale for each metabolite (each row).

Although large overall variation obscured direct visualization of inter-individual differences when using all data in PLS-DA projections, specific compounds exhibited very large concentration differences among individuals (Fig. 3). For example, the human heme-derived bile pigments biliverdin and bilirubin and the microbially produced urobilin and stercobilin varied drastically among devices for some participants and were also greatly reduced or absent in specific participants, such as stercobilin for participants 10 and 15 (Fig. 3). Production of secondary bile acids has been proposed to use a similar enzymatic pathway as stercobilin production^50,51^ and the two participants who had low stercobilin levels also showed reduced concentration of deoxycholic acid, a secondary bile acid (Fig. 3). The same participant-specific profiles of bile pigments were also observed in stool samples (Extended Data Fig. 8). Notably, these two participants (10 and 15) were the only ones who reported use of antibiotics in the 6 months before the study. These individuals were characterized by very low levels of stercobilin, deoxycholic acid, a subset of FAHFAs and sulfonolipids (Fig. 3), all of which have been previously linked to the gut microbiota^10,51–53^. Oral antibiotics can affect the intestinal microbiota for more than a year after treatment^54^. These data support the hypothesis that metabolic profiles reflect differences in microbial activity for specific pathways. We did not identify significant associations between microbial species abundance from 16S rRNA gene quantification and stercobilin levels due to limited statistical power, but the presence of FAHFAs and sulfonolipids was associated with the differential abundance of specific microbial species.

**Extended Data Fig. 9:**
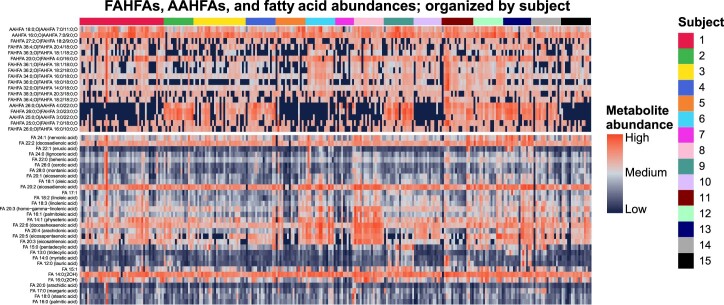
FAHFAs, AAHFAs, and fatty acids with strong inter-subject abundance differences. Metabolites are fatty acid esters of hydroxy fatty acids (FAHFAs and AAHFAs) and fatty acids detected >50% of all device samples. All device samples are shown, and are organized by subject. Within the top (FAHFA) and lower (fatty acid) sections, the metabolites are ordered based on hierarchical clustering. Color bar represents metabolite abundance (peak height) or concentration (ng/mL) for bile acids. Minimum and maximum values were used to set the color scale for each metabolite (each row).

FAHFAs were first identified 10 years ago. They are biologically active and regulate physiology^55,56^. Acyl α-hydroxyl fatty acids (AAHFAs) are a specific subset of FAHFAs with an α-lipid linkage that were discovered just 2 years ago^53^. We identified 88 FAHFAs in this study (Supplementary Table 1). Notably, four specific FAHFAs exhibited highly consistent differences among participants and all of these FAHFAs are linkages of a long-chain hydroxyl fatty acid backbone esterified with C3- or C4-short-chain moieties. Three of these bioactive lipids are esterified at the α-position (AAHFA 4:0/22:0, AAHFA 3:0/22:0 and AAHFA 3:0/24:0) and one is esterified elsewhere on the backbone (FAHFA 3:0/23:0). Nine participants frequently exhibited high levels of these FAHFAs, whereas six participants produced very little or undetectable amounts (Fig. 3). As undetectable levels cannot be used for ANOVA statistics, we did not perform significance testing. The same individual-specific trends were observed in stool samples (Extended Data Fig. 8). The only other frequently detected FAHFA with a C3 or C4 sidechain and a long-chain fatty acid was AAHFA 16:0/4:0, which did not follow the same individual-specific trend as the four FAHFAs discussed above. Of note, low levels of long-chain fatty acid or SCFA substrates, propionic (C3:0) and butyric acid (C4:0), did not explain the observed differences in these short-chain FAHFAs in intestinal or stool samples (Fig. 3 and Extended Data Fig. 9). We therefore investigated whether specific bacteria were associated with the presence or absence of the four FAHFAs of interest. Two taxa, a species in the *Blautia* genus most closely related phylogenetically to *Blautia* *obeum* and proteobacteria related to *Bilophila* *wadsworthia*, were significantly associated with detection of these FAHFAs (Supplementary Table 8). *B.* *obeum* is a known SCFA producer^57^, which suggested that production of the short-chain fatty acyl constituents might be a driver of individual-specific FAHFA production; however, FAHFA 4:0/16:0 did not show the same individual-specific trend, suggesting that the *Blautia* species may specifically produce FAHFAs with propionic and butyric acid esters of hydroxylated very-long-chain fatty acyls (22:0 and 23:0) and not of hydroxyl-forms of the most abundant fatty acids, C16–18. A chemically related group of FAHFAs have been shown to improve glucose homeostasis, stimulate insulin sensitivity and have anti-inflammatory effects^55^. Thus, our findings have potential health implications in addition to providing a link between intestinal chemistry and FAHFA levels in humans.

Here, we report the detection of sulfonolipids in human samples. These lipids were only recently added to lipidomic libraries^58^, leading to the discovery that they are microbially produced in mouse intestinal tracts and linked with both pro- and anti-inflammatory phenotypes^59–61^. In intestinal device samples, we detected sulfonolipids with strong inter-individual trends regardless of sampling time point (meals), suggesting that sulfonolipids were microbially produced in some individuals, but not in others. The two participants who had previously received antibiotics showed much lower levels of sulfonolipids than all other individuals (Fig. 3), suggesting that the antibiotic treatment may have eliminated the microbial producers. Notably, sulfonolipids were also absent in specific samples of other participants, prompting us to examine associations of sulfonolipids with bacterial taxa. Ten taxa were significantly enriched in sulfonolipid-containing samples, including a member of the Desulfovibrionaceae family (Supplementary Table 8), a family that has been associated with ulcerative colitis^62^. Also enriched were two members of Bacteroidetes, a phylum with known sulfonolipid producers^60^. Health implications of sulfonolipid levels in humans are not well understood. Sulfonolipids were recently shown to increase inflammatory responses in macrophages, but decrease inflammation in the presence of lipopolysaccharides^63^. Further investigations are required to determine the relationship of sulfonolipids with human health.

This work provides a report of metabolome differences in the upper intestinal tract in healthy human participants using a non-invasive, ingestible sampling device. Our results open the door for future detailed in vivo studies on digestion and intestinal diseases. As expected, the metabolome of stool was highly distinct from that of the intestine. Thus, stool cannot serve as a surrogate for the gut intestinal tract, rather only for colonic contents (at best). Even within the intestinal tract, >50% of annotated metabolites exhibited significantly different levels between proximal and distal locations. An important goal for future investigation is to characterize the effect of antibiotics on intestinal sulfonolipid-, stercobilin- and long-chain AAHFA-producing bacteria and the consequences of such disruptions on health and disease. The disruption of these bacteria by antibiotics may be linked to the incidence and etiology of inflammation, diabetes and inflammatory bowel disease^55,60,63^. Consequently, it will be important to uncover the dynamics and mechanisms of repopulation of antibiotic-treated individuals with these microbes.

In our related companion study^4^, we used the same device samples to broadly study variation in the intestinal environment using several omics approaches. Like the findings on metabolomes reported in this study, we found more pronounced prophage induction in intestinal samples compared to stool and that the host proteome and bile acid profiles varied along the intestines and were highly distinct from those of stool^4^. Complementing the large differences in peptides and amino acids between proximal and distal intestinal locations reported here, in our companion study we show that microbially conjugated bile acid concentrations displayed amino-acid-dependent trends that were not apparent in stool. Taken together, these studies collectively illustrate the utility of sampling directly from the intestines, which should improve our understanding of the intimate relationship between human hosts and their commensal microbes.

As a pilot study, our study has several limitations. First, a larger number of participants will be necessary for making conclusions about the range of gut metabolism within and across human populations and larger studies could also accommodate more detailed microbiome-wide associations of specific bacteria or bacterial families with metabolic conversions. Second, only two participants reported antibiotic use, hence the potential link between antibiotics use and disruption of sulfonolipid and FAHFA metabolism needs to be strengthened in future studies. Third, analysis of temporal variation was limited to two time points per day on two consecutive days, hence changes in metabolic or microbial composition over time were not fully assessed. Fourth, links between upper intestinal microbiota composition and variability to disease conditions, such as small intestinal bacterial overgrowth, will need specific, separate investigations. Despite these limitations, our findings demonstrate that the use of non-invasive sampling devices, in combination with metabolomics and genomics, has substantial potential to enable more precise intervention and prevention strategies for addressing nutritional studies and human disease.

## Methods

### Ingestible sampling device

In a related companion publication^4^, we used the same text to describe the capsule sampling device (CapScan, Envivo Bio). A CapScan consists of hollow elastic collection bladder capped by a one-way valve^4^. To prepare the device for packaging, the collection bladder is evacuated, folded in half and packaged inside a dissolvable capsule measuring 6.5 mm in diameter and 23 mm in length, onto which an enteric coating is applied. The capsule and the enteric coating are designed to prevent contamination of the collection bladder from oral-pharyngeal and gastric microbes during ingestion. The enteric coating and capsule disintegrate when the device reaches the target pH, which is pH 5.5 for type 1, pH 6 for type 2 and pH 7.5 for types 3 and type 4, with type 4 also having a time-delay coating to bias collection toward the ascending colon. After disintegration of the enteric coating, the collection bladder unfolds and expands into a tube 6 mm in diameter and 33 mm in length, thereby drawing in up to 400 µl of gut luminal contents through the one-way valve. The integrity of the sample collected inside the collection bladder is maintained by the one-way valve as the device moves through the colon and is exposed to stool.

In a related companion publication^4^, we used the same text to describe the study design. Each participant concurrently ingested sets of four devices, each with distinct coatings to target the proximal to medial regions of the small intestine (coating types 1 and 2) and more distal regions (coating types 3 and 4). After sampling, the devices were passed in the stool into specimen-collection containers and immediately frozen. After collection, the stool was thawed and devices were retrieved by study staff. For sample removal, the elastic collection bladders were rinsed in 70% isopropyl alcohol and punctured with a sterile hypodermic needle attached to a 1-ml syringe. Samples were transferred into microcentrifuge tubes and pH was measured with an InLab Ultra Micro ISM pH probe (Mettler Toledo). For metabolomics analysis, a 40-µl aliquot was spun down for 3 min at 10,000*g* to collect the supernatant. The rest of the sample was frozen until being thawed for DNA extraction.

### Study design

In a related companion publication^4^, we used the same text to describe the study design. The study was approved by the WIRB-Copernicus Group Institutional Review Board (study no. 1186513) and informed consent was obtained from each participant. Healthy volunteers were selected to exclude participants suffering from clinically detectable gastrointestinal conditions or diseases that would potentially interfere with data acquisition and interpretation.

Participants met all of the following criteria for study inclusion: (1) individuals between the ages of 18 and 70 years; (2) American Society of Anesthesiologists physical status class risk of 1 or 2; (3) for women of childbearing potential, a negative urine pregnancy test within 7 d of screening visit and willingness to use contraception during the entire study period; and (4) fluency in English, understanding the study protocol and able to supply informed written consent, along with complying with study requirements.

Participants with any of the following conditions or characteristics were excluded from the study: (1) history of any of the following: previous gastric or esophageal surgery, including lap banding or bariatric surgery, bowel obstruction, gastric outlet obstruction, diverticulitis, inflammatory bowel disease, ileostomy or colostomy, gastric or esophageal cancer, achalasia, esophageal diverticulum, active dysphagia or odynophagia or active medication use for any gastrointestinal conditions; (2) pregnancy or planned pregnancy within 30 d from screening visit or breast-feeding; (3) any form of active substance abuse or dependence (including drug or alcohol abuse), any unstable medical or psychiatric disorder or any chronic condition that might, in the opinion of the investigator, interfere with conduct of the study; or (4) a clinical condition that, in the judgment of the investigator, could potentially pose a health risk to the individual while involved in the study.

Fifteen healthy individuals were enrolled in this study and each swallowed at least 17 devices over the course of 3 d. Sample size was chosen to assess general variation across human intestinal tracts. Daily instructions included the following guidelines: record all foods and time they were consumed throughout the day; if you work out, do so in the morning; eat breakfast and lunch as usual; swallow a set of four devices 3 h after lunch with up to two-thirds cup of water; do not eat or drink anything for at least 2 h after swallowing devices; if hungry after 2 h, snack lightly (up to 200 calories); do not drink any caffeinated beverages after lunch until the next morning; collect all stool starting 6 h after swallowing this set of devices until 48 h after swallowing the next set of devices; eat dinner as usual at least 6 h after lunch; swallow a set of four CapScan devices 3 h after dinner with two-thirds cup of water; and after swallowing this set, do not eat or drink anything until the morning. Alcohol consumption and diet contents were not restricted. All ingested devices were recovered and no adverse events were reported during the study. In total, 274 capsule devices provided sufficient material for metabolomics analysis and 225 provided sufficient volume or number of sequencing reads (>2,500) for genomic analysis. Every bowel movement during the study was immediately frozen by the participant at −20 °C. Participant 1 provided additional samples for assessment of replicability. A total of 333 intestinal and stool samples were analysed with metabolomics methods.

### Untargeted metabolomics sample preparation

Sample preparation was performed using a biphasic extraction^64^ with water, methanol and methyl tert-butyl ether (MTBE) to separate polar and non-polar metabolites. Capsule device supernatant and stool samples were prepared separately because device samples were liquid and stool samples were solid. For each supernatant sample, 10 µl was aliquoted into one well of a deep sample preparation 96-well plate in a pre-determined randomized order. Samples were extracted one 96-well plate at a time and all steps were carried out at 4 °C unless otherwise specified. Between every ten experimental samples, a method blank and external QC sample were prepared. Blanks used 10 µl LC–MS-grade water instead of sample and QC samples used 10 µl pooled sample of human gastrointestinal tract contents from unrelated studies. Then, 170 µl methanol containing SPLASH LIPIDOMIX Mass Spec Standard (Avanti) were added to each well and the plate was heat-sealed with foil, shaken vigorously for 30 s at room temperature, unsealed and 490 µl MTBE was added. The plate was then heat-sealed again, vortexed vigorously for 30 s at room temperature and shaken for 5 min on an orbital shaker. The foil seal was removed and 150 µl LC–MS-grade water was added to each well. The plate was vortexed for 30 s at room temperature and centrifuged at 708*g* for 12 min. The foil was removed from the deep-well plate and two 180-µl aliquots of the top phase were transferred to two 96-well Vanquish LC plates using a 12-channel pipette. Two 50-µl aliquots of the aqueous phase were then transferred to two other 96-well Vanquish LC plates. All 96-well plates were dried completely under vacuum at room temperature, heat-sealed with foil and stored at −80 °C until further analysis. Each stool sample was prepared by mixing with a spatula and 5 ± 1 mg was transferred to a 2-ml microcentrifuge tube. Then, 225 µl methanol containing SPLASH LIPIDOMIX Mass Spec Standard (Avanti) was added to all microcentrifuge tubes and the tubes were vortexed for 10 s at room temperature. Two 3-mm stainless steel balls and 750 µl MTBE were added to each tube and samples were homogenized in a Geno/Grinder (SPEX) at 1,500 Hz for 1 min. Following that, 188 µl water was added to each tube and each tube was vortexed for 30 s at room temperature. Tubes were centrifuged at 14,000*g* for 2 min at room temperature. Two aliquots of 180 µl of the organic phase were transferred to two 96-well plates. Two 50-µl aliquots of the aqueous phase were transferred to two 96-well plates. All plates were dried completely in a rotary vacuum evaporator, heat-sealed with foil and stored at −80 °C until further analysis.

### HILIC LC–MS/MS analysis

Samples from the aqueous phase aliquots of sample preparation were removed from −80 °C and 35 µl 8:2 acetonitrile:water containing 29 isotopically labeled and synthetic internal standards, including CUDA^65^ was added to each well. The 96-well plates were then sealed with foil, vortexed vigorously for 30 s at room temperature, sonicated in a water bath for 5 min at room temperature and centrifuged for 15 min at 708*g*. Plates were stored in an autosampler at 4 °C until analysis (maximum storage time in autosampler was 48 h). LC–MS/MS was performed with a Vanquish LC (Thermo Scientific) coupled to a QExactive HF^+^ orbital ion trap mass spectrometer (Thermo Scientific). Chromatographic separation was performed with a Waters Acquity UPLC BEH Amide column (150 mm length × 2.1 mm inner diameter (i.d.); 1.7-µm particle size) with a 5-mm pre-column (5 mm length × 2.1 mm i.d.; 1.7-µm particle size). Mobile phase A was 100% water and B was 95:5 acetonitrile:water. Both mobile phases were modified with 10 mM ammonium formate and 0.125% formic acid. Flow rate was 400 µl min^−1^, column temperature was 45 °C and injection volume was 3 µl. The LC gradient was 100% mobile phase B from 0 to 2 min, 70% B by 7.7 min, 40% B by 9.5 min, 30% by 10.25 min and returned to 100% B by 12.75 min to re-equilibrate until 17 min. All mobile phase gradient shifts were linear. Acetonitrile:water 1:1 was used as a needle wash solvent before and after sample injection. Heated electrospray ionization (HESI) source conditions were as follows: sheath gas flow 50, auxiliary gas flow 13, sweep gas flow 3, capillary temperature 263 °C, S-lens RF level 50, auxiliary gas heater temperature 425 °C and needle voltage 3,500 V and −3,500 V for positive and negative ionization mode, respectively. Spectra were collected with data-dependent MS/MS acquisition (DDA) for the top four ions. MS scans were collected with 60-k resolving power from 60–900 *m*/*z*, AGC target of 10^6^ ions and maximum accumulation time of 100 ms. MS/MS spectra were collected with 15-k resolving power, 1-Da isolation window, 50-ms maximum accumulation time, a 3-s dynamic exclusion window, stepped (N)CE of 20, 30 and 60 for fragmentation and 8 × 10^3^ AGC target. All spectra were stored in centroid mode. Three rounds of iterative exclusion MS/MS were acquired for each pooled QC sample. Immediately after analysis, plates were dried under vacuum, sealed with foil and stored at −80 °C.

### Lipidomics LC–MS/MS analysis

Sample plates from the organic phase aliquots were removed from −80 °C and 50 µl 9:1 acetonitrile/toluene was added to each well. The plates were then sealed with foil, vortexed vigorously for 30 s at room temperature, sonicated in a water bath for 5 min at room temperature, centrifuged for 15 min at 708*g* and transferred to an autosampler kept at 4 °C until analysis (maximum storage time in autosampler before analysis was 48 h). Lipidomics LC–MS/MS analysis used a Thermo Scientific Vanquish LC system coupled to a Thermo Scientific QExactive HF^+^ orbital ion trap mass spectrometer. Chromatographic separation used a Waters Acquity UPLC CSH C18 column (100 mm in length × 2.1 mm i.d.; 1.7-µm particle size) with a pre-column (5 mm in length × 2.1 mm i.d.; 1.7-µm particle size). Mobile phase A was 9:1 acetonitrile:water and B was 8:2 IPA:acetonitrile. For positive-mode electron spray ionization (ESI), the mobile phases were modified with 10 mM ammonium formate and 0.1% formic acid. For negative-mode ESI, mobile phases were modified with 10 mM ammonium acetate. Flow rate was 600 µl min^−1^, column temperature was 65 °C and injection volume was 5 µl. The mobile phase gradient was 15% B from 0 to 0.6 min, 30% B by 2 min, 48% B by 2.5 min, 82% B by 11 min, 99% B from 11.5 to 12 min and 15% B from 12.1 to 14.2 min. HESI source conditions were as follows: sheath gas flow 55, auxiliary gas flow 15, sweep gas flow 3, capillary temperature 275 °C, S-lens RF level 50, auxiliary gas heater temperature 450 °C and needle voltage 3,500 V and −3,500 V for positive and negative ionization mode, respectively. DDA MS/MS spectra were acquired for the top four ions. MS scans were collected with 60-k resolving power from 120–1,700 *m*/*z*, AGC target of 10^6^ ions and maximum accumulation time of 100 ms. MS/MS spectra were collected with 15-k resolving power, 1-Da isolation window, normalized collision energy of 20, 30 and 60, 2-s dynamic exclusion window, 8 × 10^3^ AGC target and 50-ms maximum accumulation time. Spectra were stored in centroid mode. Three rounds of iterative exclusion MS/MS were acquired for each pooled QC sample. Immediately after all samples were analysed, the plates were dried under vacuum, sealed with foil and stored at −80 °C.

### Untargeted GC–MS analysis

Dried aqueous-phase samples as described above were removed from −80 °C and 10 µl 40 mg ml^−1^ methoxyamine hydrocholoride in pyridine was added to each well. The plates were sealed and shaken at 30 °C for 90 min. Foil was removed and 90 µl *N*-methyl-*N*-trimethylsilyl trifluoroacetamide (containing internal standards of C8-C30 fatty acid methyl esters) was added to each well and plates were shaken for 30 min at 37 °C. The plate was then centrifuged at 2,400*g* for 15 min at room temperature. Foil was removed and 90 µl supernatant was transferred to glass crimp-top vials with glass insert and crimped shut. Samples were analysed within 48 h of derivatization. GC–MS analysis was carried out as previously described^66^. Briefly, 0.5 µl was analysed in splitless mode through an Agilent 6890 GC equipped with a RTx-5Sil MS column (30 m × 0.25 mm i.d., 0.25 µm 95:5 dimethyl diphenyl polysiloxane film). Chromatography used helium at 1 ml min^−1^ and temperature gradient of 50 °C to 275 °C. MS analysis was performed using a Leco Pegasus III TOF mass spectrometer. Spectra were collected at 17 spectra per second with 70 eV EI. Data files were pre-processed in Leco ChromaTOF software and further analysed using the automated GC–MS data-processing pipeline BinBase^67^ with FiehnLib for spectra and retention time-matching.

### Bile acid quantification

Bile acids were targeted and quantified as previously reported^4^. Briefly, dried aqueous-phase sample plates from hydrophilic interaction chromatography (HILIC) ESI+ analysis were removed from a −80 °C freezer and dissolved in 60 µl bile acid run solvent (1:1 ACN methanol containing 100 ng ml^−1^ of five isotopically labeled bile acid internal standards). Plates were vortexed for 30 s and sonicated in a water bath for 5 min. All samples were diluted 30-fold in bile acid run solvent. All plates were centrifuged at 708*g* for 15 min at 4 °C and stored in a Vanquish autosampler at 4 °C until analysis. A nine-point standard curve was prepared between 0 to 1,500 ng ml^−1^ and analysed with samples. A Vanquish UPLC and Altis QqQ mass spectrometer (Thermo Fisher Scientific) were used for targeted LC–MS/MS analysis. An Acquity BEH C18 column (100 mm × 2.1 mm i.d., 1.7-µm particle size; Waters) was used with mobile phase A of water and B of acetonitrile, both with 0.1% formic acid. Skyline software and custom R scripts were used to process and quantify bile acid analytes.

### Short-chain fatty acid quantification

SCFA quantification was performed through derivatization with *N*-tert-butyldimethylsilyl-*N*-methyltrifluoroacetamide (MTBSTFA) based on a previously reported protocol^68^. Briefly, 10 µl intestinal supernatant was transferred to a 1.5-ml microcentrifuge tube on ice pre-filled with 50 µl LC–MS-grade water containing deuterium-labeled SCFA internal standards and 10 µl 37% hydrochloric acid. Then, 100 µl MTBE also containing deuterium-labeled SCFA internal standards was added to each tube and the tubes were shaken on rotary shaker plate for 30 min at room temperature. Tubes were centrifuged at 14,000*g* for 2 min and 20 µl MTBE (top layer) was transferred to a crimp-top GC–MS vial fitted with low recovery insert. Then, 5 µl MTBSTFA was added to each vial, which was then sealed. Vials were shaken on an orbital shaker plate for 30 min at 80 °C, cooled to room temperature and analysed by GC–MS. An Agilent 6890 GC coupled to a Leco Pegasus IV TOF mass spectrometer was used with a DB5 DuraGuard (30 m × 0.25 mm × 0.25 µm) capillary column. Following that, 1 µl was injected with 1:10 split mode enabled, helium flow rate of 1.2 ml min^−1^, temperature ramped from 50 °C to 290 °C over 20.8 min and scan rate of 17 spectra per min from 50–550 *m*/*z*. A six-point standard curve between 1 and 100 µg ml^−1^ was analysed for every 80 samples and blank samples were analysed between every ten experimental samples. Samples and standard curve mixes were prepared within 24 h of GC–MS analysis. Data were converted to Agilent (.d) format and processed using Agilent MassHunter Quant v.B.09.00. Linear six-point standard curves using ratio of analyte to internal standard were used to quantify SCFAs in experimental samples.

### Untargeted LC–MS/MS data processing

MS-DIAL v.4.80 (ref. ^58^) was used to process untargeted LC–MS/MS data. HILIC LC–MS/MS (ESI+ and ESI−) and lipidomics (ESI+ and ESI−) datasets were processed with manually optimized parameters (Supplementary Table 9). Peak height was reported for all untargeted analyses. Experimental MS/MS spectra were matched to MassBank of North America as well as NIST20 spectral libraries for HILIC analyses and all lipid reference MS/MS spectra from MS-DIAL were used for lipidomics analyses. Retention time information for HILIC and lipidomics from authentic standards run under identical chromatography conditions was used as another line of evidence for metabolite annotation. Metabolite annotations were based on accurate mass matching with retention time and/or an MS/MS match to a library spectrum. Blank subtraction was performed by retaining LC–MS features for which the maximum intensity from an experimental sample was at least ten times as high as the average of the method blanks, along with further data reduction as described in Supplementary Table 2. The final step of data processing was manual investigation of annotated features for MS/MS match quality, peak quality, peak alignment and removal of in-source fragments using correlation between features with close retention time for in-source fragment identification. When multiple metabolites from spectral libraries matched one experimental MS/MS, the match was recorded as a non-unique MS/MS match (Supplementary Table 1). Predicted retention times calculated using Retip^69^ were used as an additional line of evidence to flag low-quality annotations.

### DNA extraction and 16S rRNA gene sequence analysis

DNA was extracted using a Microbial DNA extraction kit (QIAGEN)^70^ and 50 µl from a capsule device or 100 mg stool. The 16S rRNA amplicons were generated using Earth Microbiome Project-recommended 515F/806R primer pairs and 5PRIME HotMasterMix (Quantabio, 2200410) with the following program in a thermocycler: 94 °C for 3 min, 35 cycles of 94 °C for 45 s, 50 °C for 60 s and 72 °C for 90 s, followed by 72 °C for 10 min. PCR products were cleaned, quantified and pooled using the UltraClean 96 PCR Cleanup kit (QIAGEN, 12596-4) and Quant-iT dsDNA High Sensitivity Assay kit (Invitrogen, Q33120). Samples were sequenced with 300-bp reads on a MiSeq (Illumina). Sequence data were de-multiplexed using the Illumina bcl2fastq algorithm at the Chan Zuckerberg BioHub Sequencing facility. Subsequent processing was performed using the R statistical computing environment v.4.0.3 (ref. ^71^) and DADA2 as previously described using pseudo-pooling^72^. truncLenF and truncLenR parameters were set to 250 and 180, respectively. Taxonomy was assigned using the Silva rRNA database v.132 (ref. ^73^). Samples with >2,500 reads were retained for analyses.

### Statistics and data analysis

Statistical tests were performed using R^71^. LMMs were performed using the lmerTest and lme4 R packages. To examine spatial differences across the intestine, we applied LMMs that accounted for sampling location (proximal (device types 1 and 2) or distal (device types 3 and 4)), eight food types classified from food logs (vegetable and animal protein (meat, egg and fish), grain (rice, pasta, bread and other grain), coffee or tea, dessert or alcohol (beer, wine or alcoholic seltzer), dairy and fruit) and antibiotic consumption within 6 months as fixed effect variables and inter-individual variation as a random effect variable (Supplementary Tables 3 and 4). Food types were manually assigned from participants’ written food logs using customized assessment forms. Metabolite abundances were log_10_-transformed and missing values were treated as zeros, followed by scaling from −1 and 1 before LMM analysis. A Benjamini–Hochberg^74^ correction was used to account for multiple-hypothesis testing. Correlations between the microbiota (log_2_-scaled amplicon sequence variant abundance) and metabolites (log_10_-scaled metabolite abundance) were calculated at the amplicon sequence variant-level using Pearson’s correlations. Differential abundance analysis using Limma-Voom differential abundance was utilized for FAHFAs and sulfonolipids in a presence/absence analysis. Only taxa with an absolute differential abundance >0.75 and Benjamini–Hochberg-corrected *P* < 0.1 were considered. ChemRICH^75^ was used to calculate enrichment statistics. Clustering was performed using the hclust function with the metabolite Spearman rank correlation matrix calculated using the cor function in R and Euclidean distance calculated with the as.dist function in R. PLS-DA and principal-component analysis (PCA) were performed with the ropls package in R^76^. PLS-DA models to distinguish participant and device type were assessed by sevenfold cross validation. Using 20–1,000 random permutations of class labels performed by the ropls R package to test for overfitting, models maintained Q2Y > 0.15 and *P* < 0.05 (ref. ^77^). Untargeted LC–MS/MS (HILIC and RP ESI+/−) features were normalized to the sum of internal standards for each platform, which has been shown to be more robust than normalizations to single compounds^78^. This normalization was performed by dividing each LC–MS feature by the sum of internal standard peak heights for that sample^78,79^. GC–MS data were normalized to the summed intensity of all annotated metabolites as extensively discussed in published protocols^80^. This method addresses differences specific to GC methods, recently called normalization to the total useful peak area^81^. Pooled QC data were found in a dense cluster when compared to CapScan and stool samples (Extended Data Fig. 1). During merging of datasets, metabolites detected by multiple assays were simplified to keep only data from one instrument, with preference for retaining data from the assay with lower technical variance (% coefficient of variance of pooled QC). Metabolites that were detected only in a single assay remained in the dataset, independent of the % coefficient of variance of pooled QC (Supplementary Table 1). Log_10_ transformation and zero-value imputation using one-tenth of the minimum reported peak height for non-detected features was performed for each metabolite before PCA and PLS-DA.

### Reporting summary

Further information on research design is available in the Nature Portfolio Reporting Summary linked to this article.

### Supplementary information

Supplementary Information Supplementary Figs. 1–3.

Reporting Summary

Supplementary Tables Supplementary Tables 1–9.

## Figures and Tables

**Extended Data Table 1:**
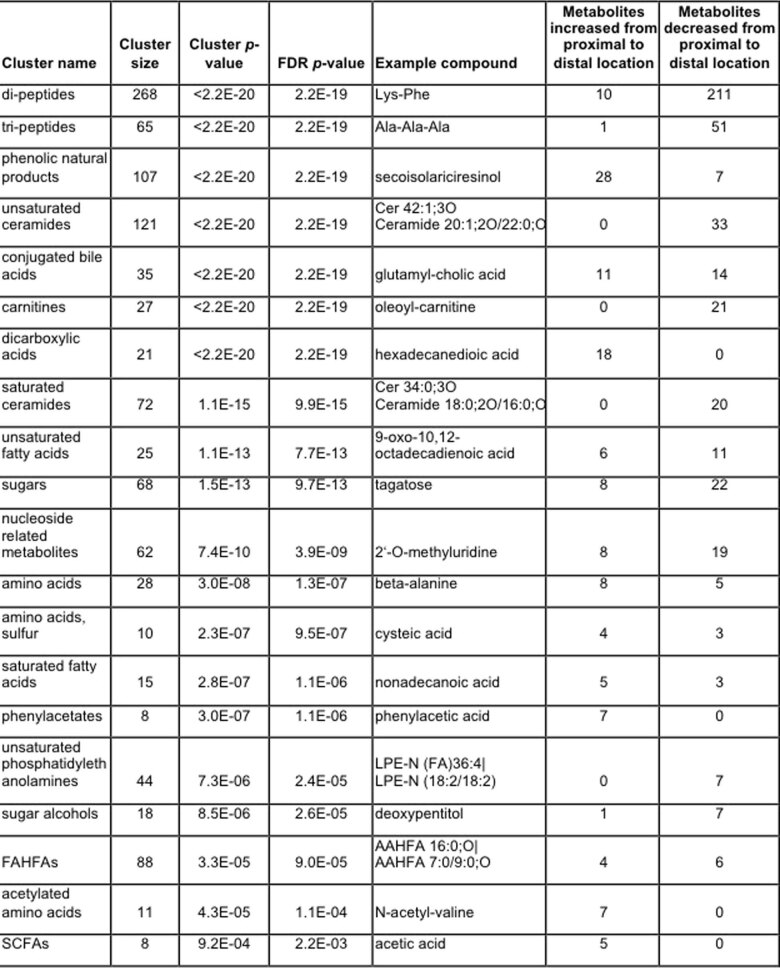
The 20 chemical classes that exhibited the most significant differences between the proximal and distal upper intestine

Significance was calculated by ChemRICH chemical enrichment statistics using univariate statistical results from an LMM as input (n = 1182 metabolites). Cluster size is the total number of metabolites within that chemical class.

## Data Availability

Raw mass spectrometry data are available on the Metabolomics Workbench (https://www.metabolomicsworkbench.org/) under studies ST002073, ST002075, ST002407, ST002409 and ST002411. The 16S and metagenomics sequencing reads are available on NCBI under BioProject PRJNA822660. Taxonomy was assigned using the Silva rRNA database v.132 (https://www.arb-silva.de/). Mass spectra were annotated using MassBank of North America public libraries (https://massbank.us/) and NIST20 libraries licensed from NIST. Scripts are archived at Zenodo (https://zenodo.org/record/7659119#.ZBGhMh_P23A).
