# Supplementary material for: Human metabolome variation along the upper intestinal tract
Source: Nat Metab. 2023 May 10;5(5):777–88. doi: 10.1038/s42255-023-00777-z (PMC10229427; doi:10.1038/s42255-023-00777-z)
Supplement: Supplementary file 1 — Supplementary Figs. 1–3. [file 42255_2023_777_MOESM1_ESM.pdf]

nature metabolism

Letter

https://doi.org/10.1038/s42255-023-00777-z

# Human metabolome variation along the upper intestinal tract

In the format provided by the authors and unedited

Supplementary data for:

## Human metabolome variation along the upper intestinal tract

Jacob Folz[1], Rebecca Neal Culver[2], Juan Montes Morales[1], Jessica Grembi[3], George Triadafilopoulos[5], David A. Relman[3,4,6,7], Kerwyn Casey Huang[4,6,8], Dari Shalon[9], Oliver Fiehn[1*]

**Affiliations:**

[1] West Coast Metabolomics Center, University of California, Davis, CA 95616, USA n

[2] Department of Genetics, Stanford University School of Medicine, Stanford, CA 94305, USA

[3] Department of Medicine, Stanford University School of Medicine, Stanford, CA 94305, USA

[4] Department of Microbiology and Immunology, Stanford University School of Medicine, Stanford, CA 94305, USA

[5] Silicon Valley Neurogastroenterology and Motility Center, Mountain View, CA 94040, USA

[6]Chan Zuckerberg Biohub, San Francisco, CA 94158, USA

[7]Infectious Diseases Section, Veterans Affairs Palo Alto Health Care System, Palo Alto, CA 94304, USA

[8]Department of Bioengineering, Stanford University, Stanford, CA 94305, USA

[9]Envivo Bio, Inc., San Francisco, CA 94107, USA

*Corresponding author: Oliver Fiehn, 451 Health Sciences Drive, Davis, CA 95616; ofiehn@ucdavis.edu

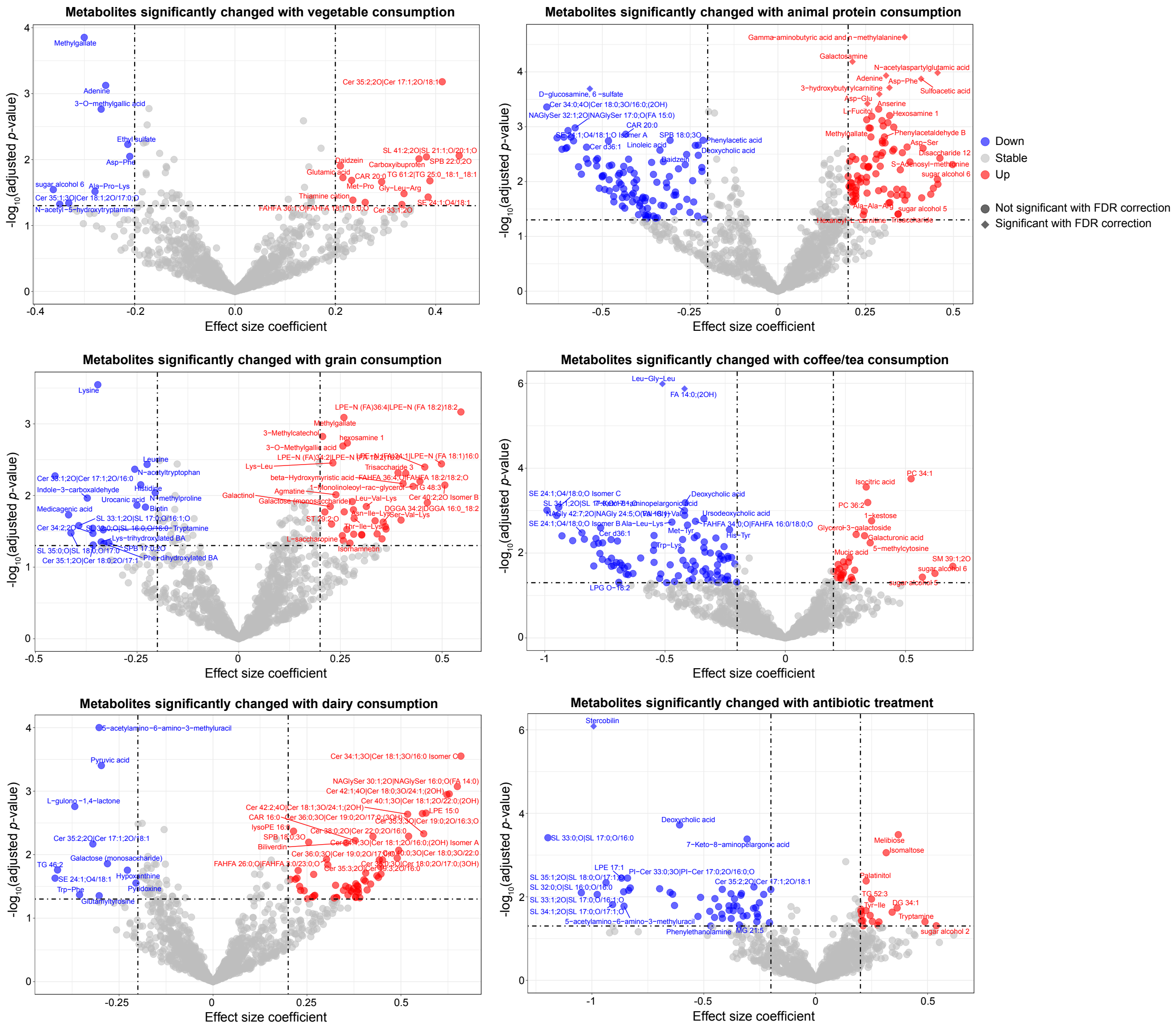


**Supplementary Figure 1. Intestinal metabolite association with food types.**
Volcano plots show significance of each metabolite to a food type (vegetable, animal protein, grain, coffee/tea, dairy, or antibiotics) calculated by linear mixed effect model (LMM). Consumption is defined to be food eaten within 6 h of ingesting sampling devices (or within 5 months for antibiotics). The dashed horizontal line represents the significance threshold of $p < 0.05$. Circles indicate non-significance after false discovery rate (FDR) correction and diamonds indicate significance after FDR correction ($n$=1182). Only metabolites detected in >50% of intestinal samples were included in this analysis. Effect-size coefficient is the slope estimated by LMM, with positive (negative) coefficient meaning the metabolite was higher (lower) after food consumption. Vertical dashed lines are ±0.2 times the effect-size coefficient.

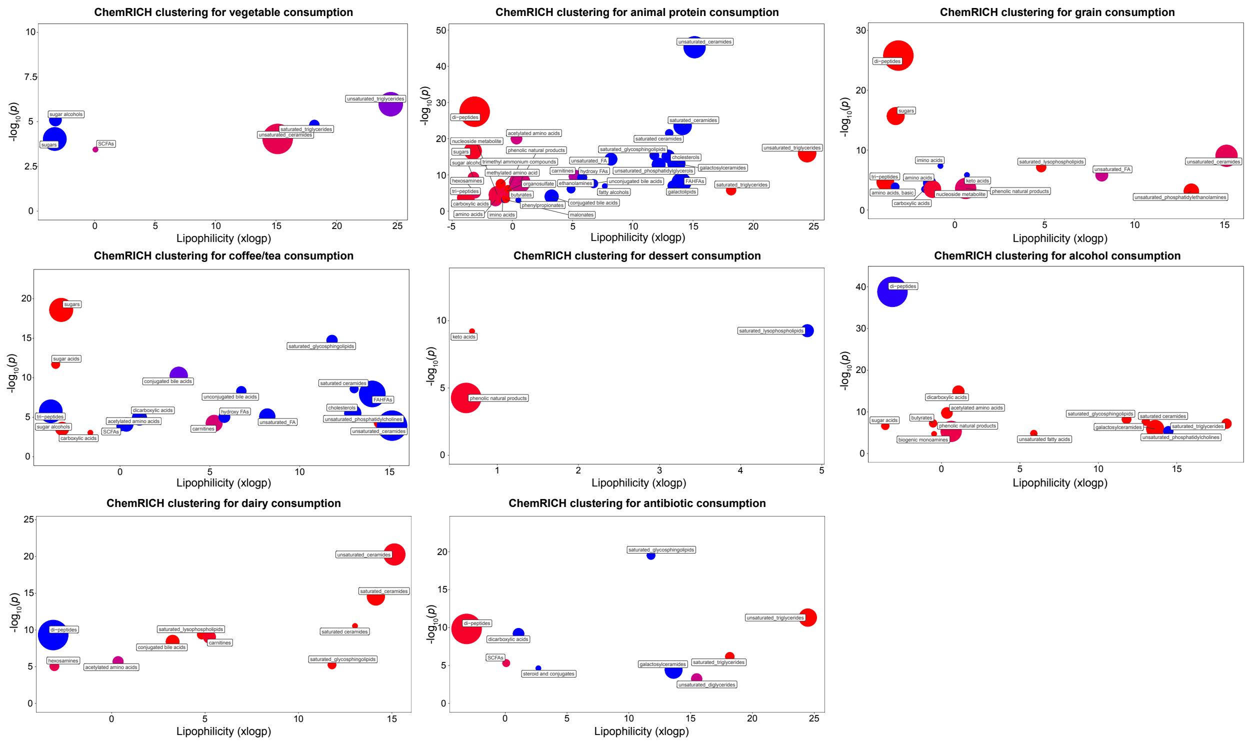


**Supplementary Figure 2.**
**Chemical classes that differed significantly after dietary intake of each food type (vegetables, animal protein, grain, coffee/ tea, dessert, alcohol, dairy, antibiotics).**
Chemical enrichment statistics were performed by ChemRICH. Only metabolites detected in >50% of intestinal samples were included in this analysis. Results were visualized by separating classes by chemical lipophilicity (logP) and chemical class significance level ($-\log_{10}(p\text{-value})$). Red (blue) circles indicate that the chemical class was higher (lower) after food consumption. Circle size represents the size of the chemical class.

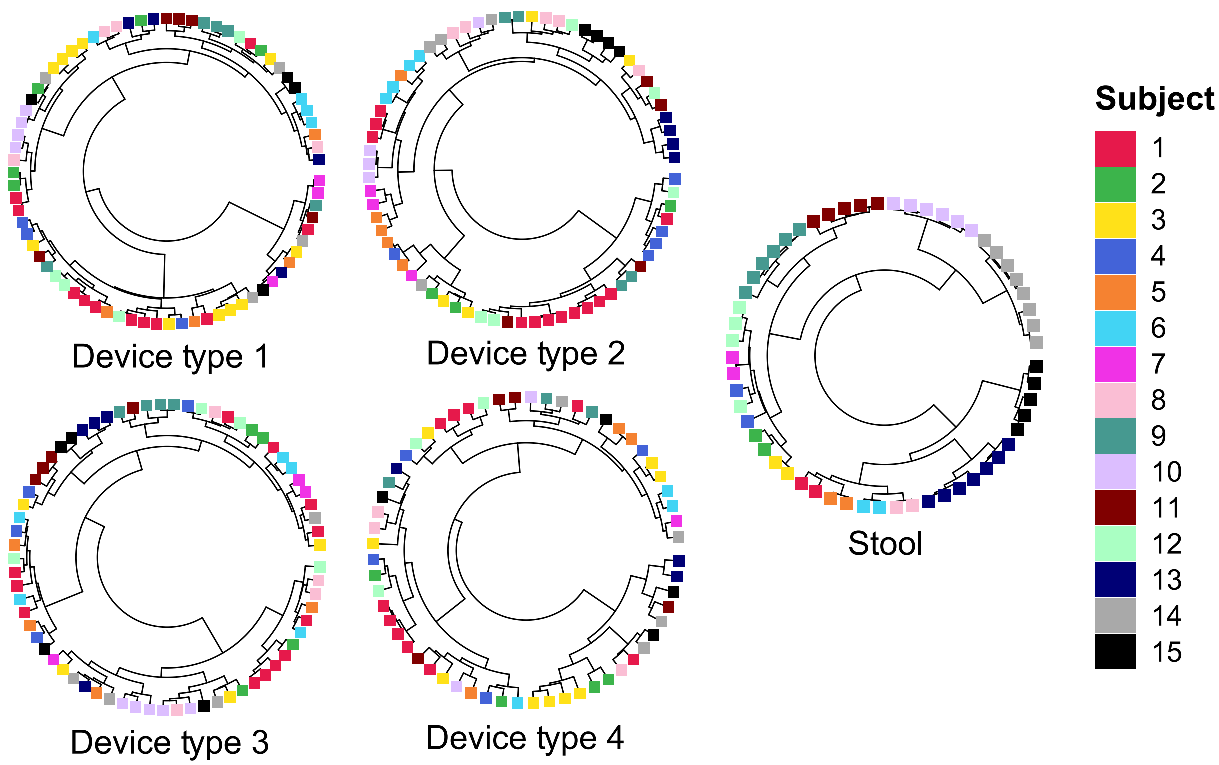


**Supplementary Figure 3. Dendrograms of sample clustering separated by sample type (device type or stool).**
Dendrograms were calculated using correlation-based hierarchical clustering of metabolites detected in >50% of device or stool samples for device type or stool dendrogram, respectively.
