# Supplementary material for: Human metabolome variation along the upper intestinal tract
Source: Nat Metab. 2023 May 10;5(5):777–88. doi: 10.1038/s42255-023-00777-z (PMC10229427; doi:10.1038/s42255-023-00777-z)
Supplement: Supplementary file 2 — Reporting Summary [file 42255_2023_777_MOESM2_ESM.pdf]

nature portfolio

Corresponding author(s): Fiehn, Oliver

Last updated by author(s): Feb 23, 2023

Reporting Summary

Nature Portfolio wishes to improve the reproducibility of the work that we publish. This form provides structure for consistency and transparency in reporting. For further information on Nature Portfolio policies, see our Editorial Policies and the Editorial Policy Checklist.

Statistics

For all statistical analyses, confirm that the following items are present in the figure legend, table legend, main text, or Methods section.

n/a	Confirmed	
☐	☒	The exact sample size (n) for each experimental group/condition, given as a discrete number and unit of measurement
☐	☒	A statement on whether measurements were taken from distinct samples or whether the same sample was measured repeatedly
☐	☒	The statistical test(s) used AND whether they are one- or two-sided<br>*Only common tests should be described solely by name; describe more complex techniques in the Methods section.*
☐	☒	A description of all covariates tested
☐	☒	A description of any assumptions or corrections, such as tests of normality and adjustment for multiple comparisons
☐	☒	A full description of the statistical parameters including central tendency (e.g. means) or other basic estimates (e.g. regression coefficient) AND variation (e.g. standard deviation) or associated estimates of uncertainty (e.g. confidence intervals)
☐	☒	For null hypothesis testing, the test statistic (e.g. F, t, r) with confidence intervals, effect sizes, degrees of freedom and P value noted<br>*Give P values as exact values whenever suitable.*
☒	☐	For Bayesian analysis, information on the choice of priors and Markov chain Monte Carlo settings
☐	☒	For hierarchical and complex designs, identification of the appropriate level for tests and full reporting of outcomes
☐	☒	Estimates of effect sizes (e.g. Cohen's d, Pearson's r), indicating how they were calculated

Our web collection on statistics for biologists contains articles on many of the points above.

Software and code

Policy information about availability of computer code

Data collection	MS-DIAL v. 4.80 was used to process untargeted LC-MS/MS data. MS/MS spectra were matched to MassBank of North America, MS-DIAL and NIST20 spectral libraries. For GC-TOF MS data, Leco ChromaTOF v4.0 was used for data acquisition and primary data processing. Compounds were annotated by FiehnLib libraries used from MassBank.us For targeted analyses of bile acids, Skyline software vs. 22.2. For targeted quantification of short chain fatty acids, Agilent MassHunter Quant v. B.09.00 was used. Retip software vs 1.0 was used for prediction of retention times. Genomic sequence data were de-multiplexed using the Illumina bcl2fastq algorithm. DADA2 software was used using pseudo-pooling with truncLenF and truncLenR parameters set to 250 and 180. Taxonomy was assigned using the Silva rRNA database v. 132.
Data analysis	Statistical tests were performed using R v. 4.0.3. Linear mixed effect models (LMM) were performed using the lmerTest and lme4 R packages. Benjamini-Hochberg was used to account for multiple-hypothesis testing. ChemRICH software vs. 1.0 was used to calculate enrichment statistics. Clustering was performed using the hclust function with the metabolite Spearman rank correlation matrix calculated using the cor function in R and Euclidean distance calculated with the as.dist function in R. Custom R scripts for metabolome data analyses are available from DOI 10.5281/zenodo.7659119 PLS-DA and PCA were performed with the ropls package in R. PLS-DA models to distinguish subject and device type had Q2Y > 0.15 and p < 0.05 determined through 20 random permutations of class labels performed by the ropls R package.

For manuscripts utilizing custom algorithms or software that are central to the research but not yet described in published literature, software must be made available to editors and reviewers. We strongly encourage code deposition in a community repository (e.g. GitHub). See the Nature Portfolio guidelines for submitting code & software for further information.

Data

Policy information about availability of data

All manuscripts must include a data availability statement. This statement should provide the following information, where applicable:

- Accession codes, unique identifiers, or web links for publicly available datasets
- A description of any restrictions on data availability
- For clinical datasets or third party data, please ensure that the statement adheres to our policy

Raw mass spectrometry data is available on the Metabolomics Workbench (https://www.metabolomicsworkbench.org/) under studies ST002073, ST002075, ST002407, ST002409 and ST002411. The 16S and metagenomics sequencing reads are available on NCBI under BioProject PRJNA822660. Taxonomy was assigned using the Silva rRNA database v. 132. Mass spectra were annotated using MassBank.us public libraries and NIST20 licensed libraries.

Human research participants

Policy information about studies involving human research participants and Sex and Gender in Research.

Reporting on sex and gender	8 women and 7 men were enrolled (gender by self determination). Enrollment targeted roughly equal distribution between men and women, which was achieved.
Population characteristics	Age mean 42, range 22-64 years. BMI mean 23, range 19-31. Details on age and BMI are given in Supplement Table S3. Two participants had taken antibiotics within the past six months. Further exclusion and inclusion criteria given in the text.
Recruitment	George Triadafilopoulos at Silicon Valley Neurogastroenterology and Motility Center, Mountain View, CA 94040, USA, recruited subjects and obtained consents and samples. Participants were all healthy community-dwelling volunteers in the local area of the study site who had first or second-hand knowledge of the study and expressed their desire to participate. We did not need to advertise in order to recruit these 15 subjects, nor did we limit recruiting to a homogeneous subject population such as graduate students. While some self-selection bias may be present in that these participants were eager to participate in the study, all subjects met the objective inclusion and exclusion criteria set out beforehand. We did not bias study participation by including or excluding subjects based on their dietary preferences. Furthermore, the subjects represented a gender balance of 8 females and 7 males, as well as a good range of ages (mean 44 years old, range 22 to 67) and BMIs (mean 23, range 19 to 31).
Ethics oversight	The study was approved by the WIRB-Copernicus Group IRB (study #1186513) and informed consent was obtained from each subject. The full name and address of the IRB that reviewed and approved the protocol is WCG IRB<br>1019 39th Ave., SE, Suite 120 Puyallup, WA 98374.

Note that full information on the approval of the study protocol must also be provided in the manuscript.

Field-specific reporting

Please select the one below that is the best fit for your research. If you are not sure, read the appropriate sections before making your selection.

☒ Life sciences ☐ Behavioural & social sciences ☐ Ecological, evolutionary & environmental sciences

For a reference copy of the document with all sections, see nature.com/documents/nr-reporting-summary-flat.pdf

Life sciences study design

All studies must disclose on these points even when the disclosure is negative.

Sample size	We chose our sample size based on the following rationale. Fifteen subjects swallowing up to 17 CapScan devices each provides a total of up to 255 device ingestions. If all subjects ingest the intended number of CapScan devices, the study has at least 80% statistical power to declare that more than 80% of the CapScan devices provided a viable liquid sample of intestinal luminal contents (that is H0: πCapScan ≤ 0.80 versus H0: πCapScan > 0.80) using the exact binomial test, assuming that at least 86.7% of the recovered CapScan devices provide a viable sample. Setting aside the extra devices ingested by subject 1, out of the 255 CapScan devices ingested by the 15 subjects, 210 CapScan devices provided sufficient liquid sample to enable DNA sequencing (see companion Nature paper preprint doi:10.1101/2022.01.19.476920), representing a sampling success rate of 82.3%. Therefore, the sample size calculation performed a priori provided adequate power to detect the observed sampling success rate. Furthermore, with 255 device ingestions, the study has at least a 95% chance of observing at least one incident of a serious device related event when the underlying incidence of the event is at least 1.17% per ingestion. The most likely serious adverse event is device retention in the intestines. Capsule endoscopy devices are retained in the intestines at a rate of around 1.4% (Gastrointest Endosc 2010, 71:280286). Capsule endoscopes are approximately twice as large as the CapScan device. We therefore assumed that the chance of CapScan retention was at most 1% and that this 15-subject first-in-human pilot study had a 95% chance of detecting this safety endpoint.
Data exclusions	no data were excluded.

Replication	This is a first study on metabolome variance in human intestinal samples. Four samples were taken for each subject, between meals during two days of this study. Reproducibility of data acquisition and data processing was ensured by using 35 GI tract pooled QC samples. Data are given in Table S1, and visualized in Extended Figure 1A. On average across the three QC batches (Table S1), metabolites showed 24% relative standard deviation as technical error. Multivariate data variance analysis and density of cluster as given in Figure 1A shows that data reproducibility was excellent, with technical errors far smaller than the biological variance in the 255 capsules plus stool samples.
Randomization	Statistical treatments were not allocated to categories or study groups. Hence, no randomization towards any study characteristic (age, BMI, sex) was needed as the purpose of the study was to find out differences between temporal and spatial variance in metabolome data. Data acquisition on mass spectrometers was fully randomized across samples and across time points or sampling locations.
Blinding	Data were collected fully blinded to age, BMI, sex or other subject-relevant covariates such as antibiotics use. We did not use age, BMI, sex or other subject-relevant covariates. Hence, statistical analyses remained blinded for subject metadata.

Reporting for specific materials, systems and methods

We require information from authors about some types of materials, experimental systems and methods used in many studies. Here, indicate whether each material, system or method listed is relevant to your study. If you are not sure if a list item applies to your research, read the appropriate section before selecting a response.

Materials & experimental systems

n/a	Involved in the study	
☒	☐	Antibodies
☒	☐	Eukaryotic cell lines
☒	☐	Palaeontology and archaeology
☒	☐	Animals and other organisms
☒	☐	Clinical data
☒	☐	Dual use research of concern

Methods

n/a	Involved in the study	
☒	☐	ChIP-seq
☒	☐	Flow cytometry
☒	☐	MRI-based neuroimaging
